# Editorial: Advancing our understanding of the cardiac conduction system to prevent arrhythmias

**DOI:** 10.3389/fphys.2025.1754225

**Published:** 2025-12-11

**Authors:** Edward Joseph Vigmond, Jason Bayer

**Affiliations:** Univ. Bordeaux, CNRS, Bordeaux INP, IMB, UMR 5251, IHU Liryc, Talence, France

**Keywords:** purkinje system, left bundle branch area pacing, cardiac conduction system, arrhythmia, ECG

## Introduction

The cardiac conduction system is responsible for the heart’s rapid activation and is vital for its optimal functioning. Its ventricular components were only discovered and satisfactorily explained at the beginning of the 20th century when Tawara meticulously traced the cardiac conduction system (CCS) from its origin at the atrioventricular node to the Purkinje fibres, which had been described some 70 years earlier but with an unknown function. Its fine and integrated structure prevents it from being seen in its entirety by the naked eye, as well as limits the magnitudes of the electrical signals produced by it. However, despite its small mass, the CCS greatly affects propagation through myocardial tissue. Even during non-sinus activation, the CCS is activated retrogradely, and exerts its influence on activation (Pollard et al., 1997).

Dysfunction of the CCS has serious consequences. Pathologies involving it can lead to arrhythmias through several mechanisms (Haissaguerre et al., 2016); the CCS can be a source of ectopic beats which interact with the sinus beat to produce reentry (Deo et al., 2010); it can help sustain arrhythmias by providing escape conduction pathways to allow wave propagation to continue (Bayer et al., 2022); finally, unidirectional block within the CCS can lead to CCS reentries (Wald et al., 1980).

It is the goal of this Research Topic to elucidate how the CCS may contribute to arrhythmias, how to identify CCS involvement when presented with an arrhythmia, as well as how to repair/manipulate the CCS to restore heart function. In this Research Topic, several research teams explored these questions from the basic science level, to the clinic using state-of-the-art biological experiments, clinical measurements, as well as computer modelling.

### Basic science

In his review of Purkinje systems and their calcium handling, Stuyvers (*Calcium arrhythmogenicity of Purkinje fibers: importance of the animal model*) argues that large animal models are appropriate for human translation, showing the same mechanisms for arrhythmogenic behaviour in the ischemic and post myocardial infarction heart (ref the paper). For small animals, like mice, changes in gene expression may mimic those occurring in humans under these conditions.

Trying to gain insight for human disease, Vahdat et al. (*Conduction defects and arrhythmias in mdx mice are not associated with a degeneration of the cardiac Purkinje network*) examined conduction disturbances in mice with Duchenne muscular dystrophy. They performed experiments to conclude that the Purkinje system was not affected and conduction disruption was due to fibrosis and reduced sodium current. As Stuyvers argues, these results are translatable to humans.

The exact structure of the CCS is difficult to image in its entirety, given the lack of CCS specific contrast agents and its small mass with fine structures. Furthermore, the electrical signals produced by it are small in magnitude. In light of this, Boukens et al. (*Functional conduction system mapping in sheep reveals Purkinje spikes in the free wall of the right ventricular outflow tract*) further clarified whether Purkinje fibres were present in the right ventricular outflow tract, a region thought to be highly arrhythmogenic (Aras et al., 2022). They recorded signals corresponding to Purkinje activations and staining for connexon 40, a CCS gap junction protein, was positive, demonstrating the presence of the CCS. This has important implications for future treatment of arrhythmias arising from this region.

### Clinical

ECG analysis cannot be performed without considering the role of the CCS, whether in a healthy or pathological state. The distribution of pulses by the CCS is reflected in the QRS for anterograde activation, and during arrhythmias, it also carries out retrograde atrial activation. Thus, for accurate diagnosis, the role of the CCS is vital, whether it be a source of ectopy, have regions of localized block, or be healthy. This is illustrated in two examples.

Wang et al. (*Case Report: Adenosine-induced atrioventricular dissociation: unmasking monomorphic tachycardia as a diagnostic challenge in a neonate*) removed the influence of the atrioventricular node to improve diagnosis in a neonate. By inhibiting the initial component of the CCS, the atrioventricular node, they blocked propagation of atrial signals into the ventricles and convincingly demonstrated that the arrhythmia continued in the ventricles and was a ventricular tachycardia.

Li et al. (*The λ pattern on time-RR interval scatter plot of neonatal ambulatory ECG: a marker of transient bradycardia*) defined a new rhythm based on time-RR interval scatter plots in neonates. They identified junctional escape rhythms (which often originate in the atrioventricular node or other parts of the CCS) and the gradual return to normal sinus rhythm. Again, the CCS is integral to understanding what is happening, so that the proper treatment may be employed.

### Modelling

Due to the fine and integrated structure of the CCS, along with its small mass yielding weak electrical signals, investigating the electrophysiology of both the healthy and pathological states of the CCS in three-dimensions is challenging using the basic science and clinical approaches discussed above. Therefore, computer modelling is used to investigate the CCS in its entirety with both high spatial and temporal resolutions. This allows investigators to test a wider range of hypotheses for the structure/function of the CCS, as illustrated in the following three examples.

Jabbour et al. (*Acute ischaemia and gap junction modulation modify propagation patterns across Purkinje-myocardial junctions*) investigated an unexpected observation that rabbit ischemic right ventricular preparations activated in less time than healthy hearts. By combinging experiments with computer modelling, they suggest that more Purkinje-myocyte junctions become functional during ischemia.

The CCS architecture influences the activation of the ventricles, but whether it also influences reentry is unclear. Bayer et al. (*To reconnect or not reconnect distal Purkinje fibers, that is the question when modelling the Purkinje fiber network*) show in a biophysically-detailed three-dimensional human ventricles model that representing the Purkinje network with a simple tree-like branching structure as opposed to a mesh, results in similar sinus activation, but different retrograde activation and reentry behaviours. This highlights the need for accurate modelling of the CCS, particularly for ectopic foci and pacing studies when activation originates from the ventricular myocardium.

From a more clinical perspective, Strocchi et al. (*Comparing the effects of left bundle branch pacing and leadless right ventricular pacing on intraventricular and interventricular dyssynchrony using in silico modelling*) used a similar modelling methodolgy to investigate the efficacy of resynchronizaton therapies, specifically leadless right ventricular pacing versus left bundle branch pacing. They showed that under most circumstances, directly stimulating the proximal left bundle branch leads improved ventricular synchrony.

## Conclusion

The CCS profoundly affects the propagation of electrical signals into and within the ventricles. It is directly responsible for the PQ interval and the shape of the QRS on the ECG which is the major diagnostic tool of cardiology. Treatment of pathologies involving the CCS include ablation of ectopic sources (Nogami et al., 2023), denetworking (Sciacca et al., 2022), and resynchronization therapy (Joza et al., 2025). Indeed, His and left bundle branch area pacing have recently revolutionized cardiac resynchronization therapy (Yeshwant and Upadhyay, 2025).

This Research Topic has contributed to the development of the above by demonstrating that mice and sheep are good animal models for understanding CCS abnormalities, elucidating functional aspects of CCS activation through modellingand experiments, helping to distinguish CCS diseases seen in the ECG, and determining the most optimal pacing protocols for restoring heart function. As we understand more about the CCS, we will be in a better position to further exploit it for preventing life threatening arrhythmias and restoring normal heart function.
